# Lack of Catch-Up Growth with Growth Hormone Treatment in a Child Born Small for Gestational Age Leading to a Diagnosis of Noonan Syndrome with a Pathogenic *PTPN11* Variant

**DOI:** 10.1155/2021/5571524

**Published:** 2021-06-07

**Authors:** Daniel J. Olivieri, Lauren J. Massingham, Jennifer L. Schwab, Jose Bernardo Quintos

**Affiliations:** ^1^The Warren Alpert Medical School of Brown University, Providence, RI, USA; ^2^Division of Genetics, Hasbro Children's Hospital/Warren Alpert Medical School of Brown University, 593 Eddy Street, Providence, RI 02903, USA; ^3^Division of Pediatric Endocrinology and Diabetes, Hasbro Children's Hospital/Warren Alpert Medical School of Brown University, 593 Eddy Street, Providence, RI 02903, USA

## Abstract

**Background:**

Growth hormone (GH) treatment increases the adult height of short children born small for gestational age (SGA). Catch-up growth is associated with a younger age, shorter height, and prepubertal status at the onset of GH treatment. We report a 12 11/12-year-old girl born SGA who received GH for 5 years without catch-up growth and was diagnosed with Noonan Syndrome (NS).

**Results:**

A 5-year-and-9-month-old 46, XX girl born SGA was started on GH treatment at a dose of 0.32 mg/kg/week. Her midparental target height is 158.6 cm. Endocrine work up showed an IGF-1 level 69 ng/ml (Normal (N): 55–238 ng/ml), IGFBP3 2.6 mg/L (N: 1.9–5.2 mg/L), TSH 3.2 mIU/L (N: 0.35–5.5 mIU/L), and a normal skeletal survey. Height was 96 cm (0.1%; Ht SDS −2.9), weight 14 kgs (1%; Wt SDS −2.3), and Tanner 1 breast and pubic hair were observed. Due to the poor catch-up growth on GH treatment, she was referred to Genetics to elucidate genetic or syndromic causes of short stature. She was noted to have posteriorly rotated ears and slight down slanting of the palpebral fissures. Genetic findings showed a heterozygous pathogenic variant in *PTPN11* (c.922A > G (p.Asn308Asp)) diagnostic for NS. This finding is de novo given negative parental testing. She was noted to have a heterozygous missense variant of unknown significance (VUS) in *FGFR3*: c.746C > A (p.Ser249Tyr). *FGFR3* is associated with multiple skeletal dysplasias including thanatophoric dysplasia, achondroplasia, and Crouzon syndrome and hypochondroplasia. Clinical correlation is poor for these syndromes.

**Conclusion:**

Diminished catch-up growth and response to GH treatment in a child born SGA led to the diagnosis of NS. The concomitant diagnosis of SGA and NS may have affected the responsiveness of this child to the growth promoting effect of GH treatment.

## 1. Introduction

Small for gestational age (SGA) has a global incidence of 3–10% and results in a higher risk for metabolic dysregulation and reduced fetal growth [1, 2]. While over 85% of infants will “catch-up” to their peers by two years of age, nearly 10% of SGA infants will continue to fall below the 3rd percentile of height until adulthood [3]. Failure to “catch-up” to peers—children two standard deviations below the mean for age and sex at two years of age per Food and Drug Administration criteria or four years of age per European Medicines Agency criteria—can warrant Growth Hormone (GH) pharmacologic treatment [4, 5]. Nonresponders to GH treatment warrant complete metabolic and genetic workup to determine the etiology [6–9]. We present a patient born SGA who was refractory to GH treatment and later found to have an *FGFR3* variant of unknown significance (VUS) and a PTPN11 pathogenic variant of Noonan Syndrome (NS). Initial clinical presentation, serial measurements, workup, and case management are discussed.

## 2. Case Presentation

The patient initially presented to the pediatric endocrinologist at Hasbro Children's Hospital (Providence, RI, USA) as a five years and two months old female for growth evaluation.

She was born to a 32-year-old G1P0 ⟶ 2 mother at 37 weeks, 2 days gestation, via cesarean section and her twin brother (discordant) during an uncomplicated pregnancy. Her birth was notable for intrauterine growth restriction (IUGR) with weight and length more than two standard deviations below the mean (1.75 kg and 38.1 cm length). The perinatal course included a 1-week hospital stay for catch-up growth but no NICU stay. Her twin brother was born at 5 pounds, 1 ounce, and 18 inches length. Maternal medications included levothyroxine, and the mother's past medical history was notable for papillary thyroid cancer. No drugs, alcohol, or cigarettes were reported nor documented in pregnancy. She required early intervention at 6 months for a motor delay (inability to sit up). Her predicted midparental target height is 157 cm ± 10 cm (62 inches ± 4 inches) with a lower limit of 147 cm (58 inches).

A concern for failure to catch up in growth by age four years led to a referral to the pediatric endocrine clinic at five years and two months of age. Her parents denied polyuria, polydipsia, headache, fatigue, fever, weight loss, cold intolerance, sleep disturbance, abdominal pain, and constipation.

The initial laboratory investigation was normal (Table 1). The physical examination findings included a height of 96.1 cm (0.2%; SDS of −2.9), a weight of 14.0 kg (1%; SDS of −2.3), and a BMI of 15.1 kg/m^2^ (50.3%). She had Tanner 1 breast and pubic hair and otherwise unremarkable physical examination. Given the height of 2.9 standard deviations below the mean at age 4 years, GH treatment was warranted [10]. She began recombinant growth hormone at a dose of 0.6 mg administered subcutaneously every night at a starting dose of 0.32 mg/kg/week.

**Figure 1:**
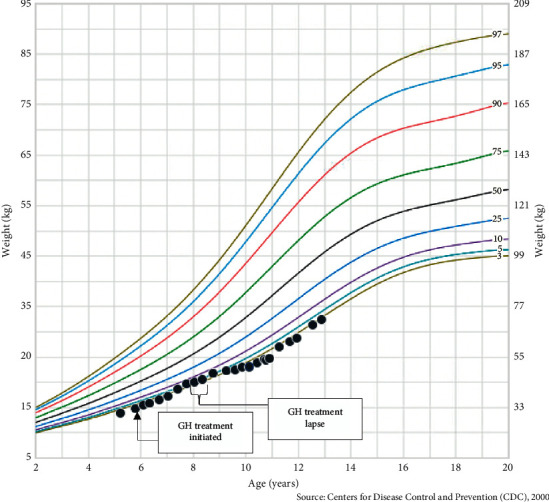
Plotted weight-for-age percentiles for Jane Doe. Centers for Disease Control and Prevention (CDC) weight-for-age percentiles, girls 2 to 20 years old.

**Figure 2:**
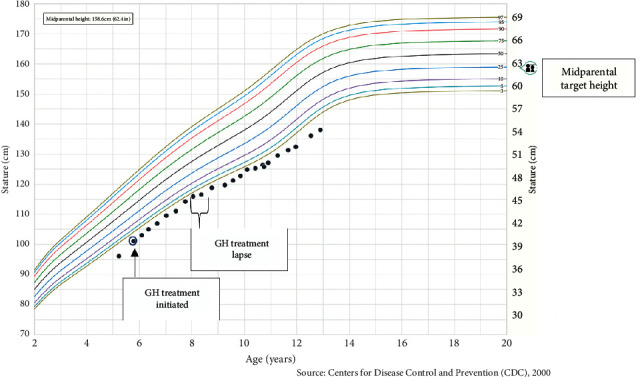
Plotted stature-for-age percentiles for Jane Doe. Centers for Disease Control and Prevention (CDC) stature-for-age percentiles, girls 2 to 20 years old.

A six-month follow-up visit was notable for an excellent annualized growth rate of 10.4 cm/year. GH dose was increased from 0.6 mg SQ to 0.65 mg SQ every night to maintain a dosage of 0.32 mg/kg/week. She denied side effects of GH injections including symptoms of pseudotumor cerebri, such as headaches, blurry vision, or vomiting. The subsequent annual growth velocities and height standard deviations (Ht SDS) are presented in Table 2. Additionally, there was a 9-month treatment lapse from years two, three due to financial cost (i.e., high health insurance deductible), resulting in diminished growth (Figures 1 and 2). GH therapy treatment was reinitiated at an age of 8 years.

Due to the continued diminished growth, she was referred to the Division of Genetics at 10 years and 9 months of age to elucidate genetic or syndromic causes of short stature. She was noted to have posteriorly rotated ears and slightly downward slanting of the palpebral fissures. A comprehensive Short Stature Syndrome Panel Plus (sequencing and deletion/duplication) from Blueprint Genetics analyzed 374 genes including IGF-1, IGF-1 receptor, IGF-ALS (insulin-like growth factor-binding protein, acid-labile subunit), GH, GH receptor, GH releasing hormone receptor, and STAT5b demonstrated a heterozygous pathogenic variant in *PTPN11* (c.922A > G (p.Asn308Asp)) diagnostic for NS. This finding is de novo as parental testing for the *PTPN11* variant was negative. She was noted to have a heterozygous missense variant of unknown significance (VUS) in *FGFR3*: c.746C > A (p.Ser249Tyr). *FGFR3* is associated with multiple skeletal dysplasias including thanatophoric dysplasia, achondroplasia, Crouzon syndrome, and hypochondroplasia. Clinical correlation is poor as our patient's findings are not as severe as typically described for these syndromes. The patient then received comprehensive screening for NS, including a renal ultrasound, EKG, skeletal survey, ophthalmic evaluation, and thyroid panel.

At the last endocrine visit at 12 years and 11 months of age, the patient's height was 138 cm (0.4%; SDS = −2.6) and weight was 32.4 kg (2.3%; SDS = −2.0) on 2.2 mg/day subcutaneous GH treatment injections (0.5 mg/kg/week). Additionally, she was noted to have Tanner 3 breast development and Tanner 4 pubic hair. Other notable physical examination findings included frontal bossing and triangular facies. Her most recent annualized growth was 5.2 cm/year (69%; SDS = 0.52). Her most recent bone age was 11 years–11 years and 6 months, with a predicted adult height of148.5 cm–150 cm vs. the midparental target height is 157 cm ± 10 cm (62 inches ± 4 inches) with a lower limit of 147 cm (58 inches). The plan is to discontinue GH treatment once the bone age is 14-15 years and growth rate is <2.5 cm/year.

## 3. Discussion

We report a 12 11/12-year-old girl born SGA who received GH for 5 years without catch-up growth and was diagnosed with NS with a *PTPN11* pathogenic variant. The concomitant diagnosis of SGA and NS may have affected the responsiveness of this child to the growth-promoting effect of GH treatment.

Children born SGA require careful follow-up and monitoring to evaluate for developmental and growth-related complications [10]; children born SGA on average weigh less and are shorter than their non-SGA counterparts [1, 11]. While the majority of SGA infants experience “catch-up” growth, nearly 15% of SGA infants will not “catch-up” by two years of life [12]. Children who remain two or greater standard deviations below the mean for their age and sex warrant consideration for GH treatment [13]. The efficacy of GH treatment in children born SGA has been correlated to earlier treatment initiation, shorter starting height, and decreased starting weight [14]. Important considerations for GH treatment include financial cost, treatment compliance, and the psychological effects of short stature [15–17]. Despite the strong efficacy of GH treatment associated with children who fit FDA or EMA criteria, some children will remain two standard deviations below their midparental target height refractory to GH therapy [18]. Potential causes of children refractory to GH include mutations in the GH receptor, IGF-1 gene, and GH signaling pathway [19]. Specifically, in regard to children born SGA, variability in response to GH treatment has been reported and may be explained through genetic mutations in the GH/GH receptor transduction signaling pathway [19, 20]. More recent studies analyze the role of growth-plate-related genes including COL2A1, COL11A1, ACAN, and FLNB [21, 22]. GnRH agonists may be indicated in children born SGA who are in puberty with predicted adult height 2.5 standard deviations below the mean for their corresponding age and sex [23]. Some children born SGA who are short at the start of puberty may benefit from the combined use of GH and GnRH agonist treatment [23–25].

NS is a genetic condition associated with short stature and is inherited in either an autosomal dominant or de novo (sporadic variant) manner [26]. The incidence of NS is approximately 1 in 1,000–2,500 births. Besides short stature, it is also commonly associated with cardiac abnormalities, characteristic facies, lymphatic dysplasias, and coagulation defects [26–28]. Studies have shown that, in children with NS, prepubertal GH treatment has resulted in increased growth at a rate equivalent to girls with Turner syndrome, although not to the level of children with idiopathic GH deficiency [27, 28]. The thought is there is a possibly impairment of the GH IGF1 axis. Therefore, GH treatment is an FDA-approved indication for children diagnosed with NS, with nearly 60% of children with NS obtaining an adult height within one standard deviation of their midparental target height [29]. Without GH treatment, nearly 70% of children with NS will have short stature throughout adulthood [29, 30]. Side effects from GH treatment in children with NS are infrequently reported. Most notable in regard to NS, GH treatment does not influence cardiac morphogenesis [29, 31]. One potential mechanism for the GH resistance in NS relates to the *PTPN11* variant. *PTPN11* encodes SHP2, a protein tyrosine phosphatase involved in GH signaling [20, 32]. Therefore, postreceptor GH signaling mutations in SHP2 might explain the mild resistance to GH treatment sometimes seen in NS patients [32]. Additionally, the downstream signaling mutation in SHP2 might also explain the compensatory elevated GH secretion occasionally seen in NS patients [32]. Finally, although recombinant IGF-1 therapy may be a potential treatment in children with NS, to the best of our knowledge, there have been no reports showing the efficacy of IGF-1 in children with NS [33].

Our patient born SGA was also diagnosed with *PTPN11*-related NS after extensive genetic testing. Additionally, genetic testing was not diagnostic for other conditions that could explain her short stature, including GH receptor, IGF-1 gene, or GH signaling pathway abnormalities. While children born SGA and children with NS can have significant increases in growth with GH treatment [34], our patient had poor response to GH treatment. Therefore, we hypothesize that the concomitant diagnosis of SGA and *PTPN11*-related NS may have affected the responsiveness of our patient to the growth-promoting effect of GH therapy. Additionally, it is important to note that genetic workup also elucidated a nondiagnostic VUS in the FGFR3 gene (c.746C > A (p.Ser249Tyr)). While pathogenic variants in this gene can be associated with several genetic syndromes including thanatophoric dysplasia, achondroplasia, Crouzon syndrome, and hypochondroplasia, our patient's phenotype did not fit these diagnoses, and at this time, the significance of this molecular finding is still unclear.

## 4. Conclusions

We present a case of a 12 11/12-year-old girl born SGA who received GH for 5 years without catch-up growth and was later diagnosed with NS. In turn, the concomitant diagnosis of SGA and NS may have altered the child's ability to respond to GH treatment.

## Figures and Tables

**Table 1:** Initial laboratory values.

Description	Value	Reference range
Genetic		
Karyotype	46, XX	—

Thyroid markers		
TSH, high sensitivity	2.977 uIU/mL	0.350–5.5 uIU/mL
Thyroglobulin Ab	<15.0 U/mL	0.0–60.0 U/mL
Thyroid peroxidase Abs	<28.0 IU/mL	1.0–60.0 IU/mL

Endocrine		
IGF-1 binding protein 3	2.6 mg/L	1.1–5.2 mg/L
IGF-1	69 ng/mL	55–238 ng/mL

Renal		
Ultrasound	Normal findings	—

Inflammatory markers		
Celiac disease	Negative	—

**Table 2:** Annual growth parameters.

Year	Height standard deviations^a^ below mean for age and sex	Growth rate (cm/year)
0	−2.9	—
1	−2.5	7.5
2	−2.2	6.7
3	−2.2	4.5
4	−2.2	5.1
5	−2.5	5.1

^a^Height standard deviations refers to the number of standard deviations below the mean height for age and sex.

## Data Availability

No data were used to support this study.
